# Multifocal lymphangioendotheliomatosis with thrombocytopenia: Report of a case with favorable outcome into adulthood

**DOI:** 10.1016/j.jdcr.2024.04.045

**Published:** 2024-05-11

**Authors:** Hasna Kerrouch, Laurie Gouillon, Remi Duclaux-Loras, Carole Burillon, Jean Kanitakis, Naoufal Hjira, Laurent Guibaud, Denis Jullien

**Affiliations:** a Department of Dermatology, Faculty of Medicine Lyon-Est, Edouard Herriot Hospital, Lyon, France; b Dermatology Venerology Department, Military Hospital Instruction Mohammed V, University Mohammed V, Rabat, Morocco; c Claude Bernard Lyon 1 University, Villeurbanne, France; d Department of Pediatric Hepatogastroenterolgy and Nutrition, Femme-Mère-Enfant Hospital, Hospices Civils de Lyon, Bron Cedex, France; e Department of Ophthalmology, Edouard Herriot Hospital, Hospices Civils de Lyon, Lyon, France; f National Referring Center for Superficial Vascular Anomalies, Hôpital Femme Mère Enfant, Bron Cedex, France

**Keywords:** angiomatosis, multifocal lymphangioendotheliomatosis, thrombocytopenia

## Introduction

Multifocal lymphangioendotheliomatosis with thrombocytopenia (MLT) was initially described in 2004 by Norton et al.^1^ To date, approximately 44 cases have been documented in the medical literature. MLT is a rare congenital disease characterized by the presence of multiple vascular skin lesions present from birth, which manifest as oval-to-round reddish-brown papules or plaques, accompanied by visceral involvement and severe thrombocytopenia.^2^ Frequently, patients with MLT experience life-threatening gastrointestinal (GI) involvement, particularly in the neonatal period.^3^ Less frequently, other organs are affected, including the central nervous system (CNS), lungs, kidneys, muscles, bones, and spleen.^4^ The diagnosis relies on clinical and pathological features, including positive lymphatic markers. Due to its rarity, no standard treatment exists, and there is limited data on long-term prognosis. To the best of our knowledge, there are rare reports of MLT in adult patients with ophthalmic involvement. We present a case of a patient with MLT with ophthalmic involvement and a favorable long-term outcome into adulthood.

## Case report

**Fig 1:**
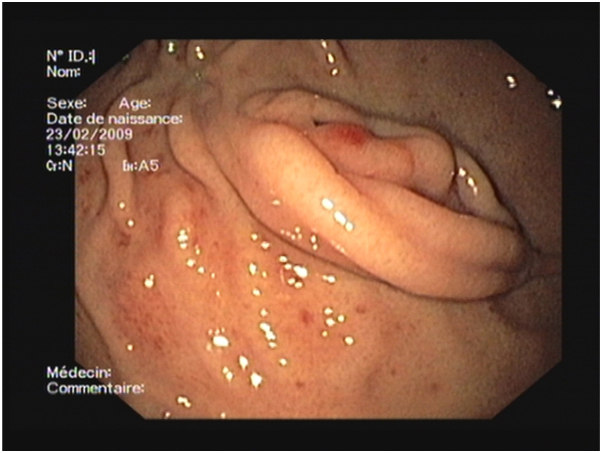
Gastrointestinal endoscopy revealing multiple mucosal vascular lesions and bleeding throughout the stomach and colon.

**Fig 2:**
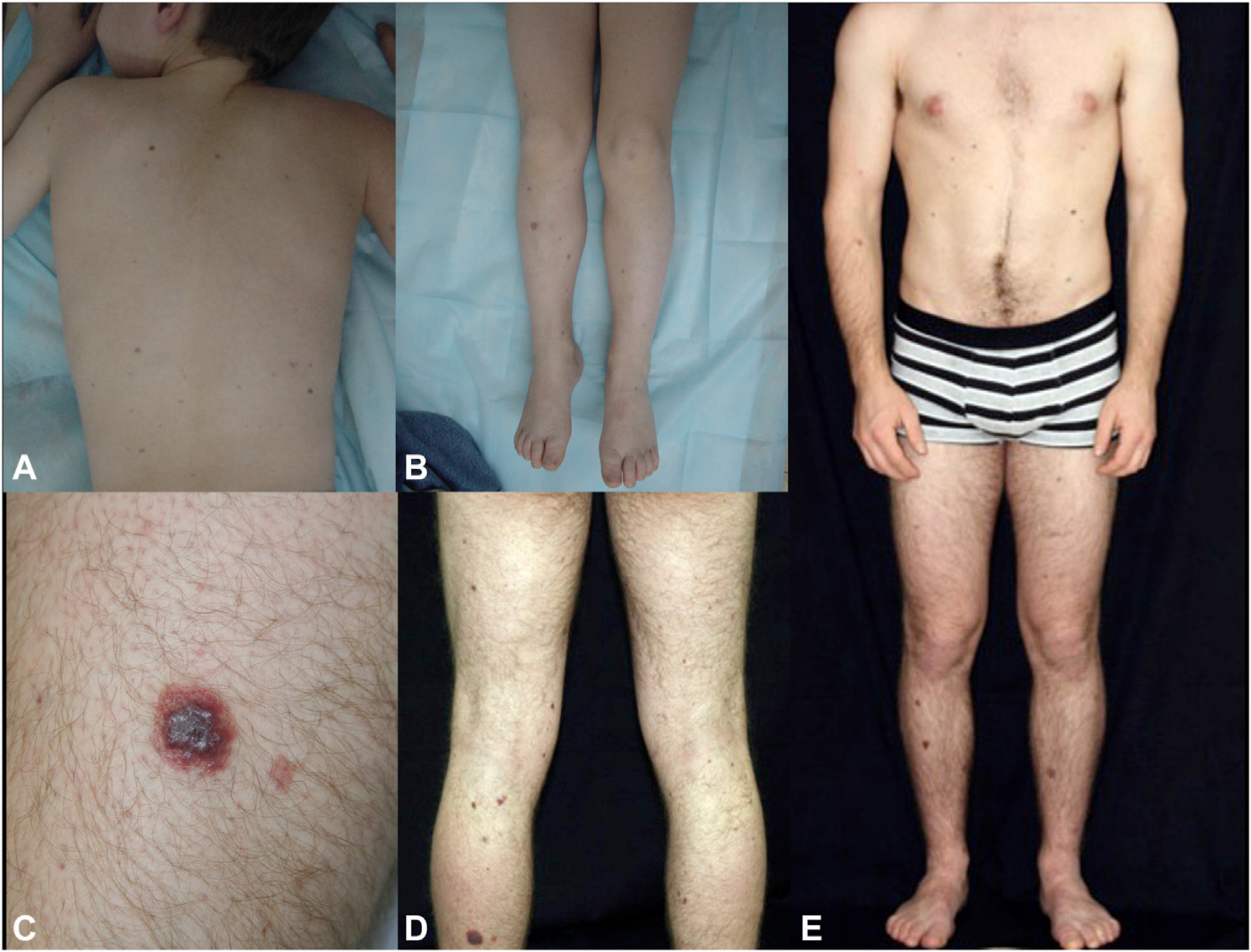
Clinical features: widespread erythematous-violaceous macules with numerous firm nodules on the limbs, trunk, and lower extremities during childhood (**A** and **B**); diffuse erythematous-violaceous macules and erythematous-violaceous nodule on the anterior of the left leg in adulthood (**C**-**E**).

**Fig 3:**
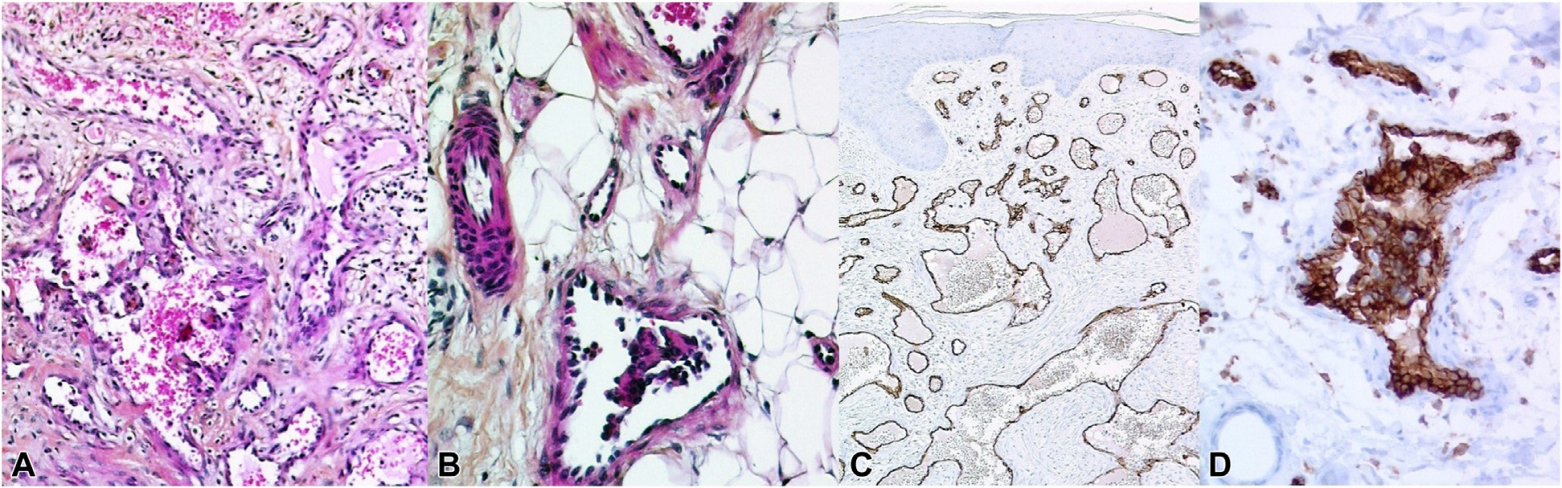
Histopathological examination shows a diffuse dermal-hypodermal vascular proliferation, made of dilated thin-walled vessels of variable size (**A**) and slightly hobnailed endothelial cells with intravascular papillary projections (**B**). Immunohistochemical staining reveals positivity of CD34 (**C**) and CD31 (**D**).

The patient was born at term to healthy parents following an uneventful pregnancy. He was the fourth child in a family without a history of cutaneous, genetic, or bleeding disorders. Within the first month of life, he developed upper GI bleeding with melena and thrombocytopenic purpura (platelet count of 30,000/μL) and required platelet and blood transfusions. GI endoscopy and colonoscopy revealed multiple mucosal bleeding vascular lesions throughout the stomach and colon (Fig 1). Gastric and intestinal biopsies confirmed diffuse GI angiomatosis. At 1 month of age, the patient underwent hemostatic partial gastrectomy. He was treated with systemic corticosteroids (2-4 mg/kg/d during episodes of bleeding or thrombocytopenia) from age 1 to 6 months and subsequently with interferon-alpha from age 6 months (subcutaneous injections of 3 × 10^6^ IU/m^2^ 3 times a week initially, followed by 10^6^ IU/m^2^ once daily), which allowed a reduction in systemic corticosteroid dosage. Interferon-alpha injections were gradually tapered and ceased at 2 years of age. Recurrent GI tract bleeding and thrombocytopenia (40-110,000 cells/μL) necessitated multiple hospitalizations and blood cell transfusions. Systemic corticosteroids were gradually tapered and discontinued at 7 years of age. The patient underwent argon plasma coagulation and ethanol sclerotherapy for recurrent GI bleeding at ages 5 and 6 years. Imaging follow-up, including cerebral magnetic resonance imaging, abdominal ultrasonography, and thoracic computed tomography, yielded normal results. Bone X-rays revealed multiple abnormalities indicative of intraosseous angiomas. Ophthalmic examination identified 1 retinal and 2 iris angiomas, one of which caused vitreous hemorrhage necessitating vitrectomy of the right eye. Thrombocytopenia resolved at age 12, allowing for the discontinuation of platelet transfusions. At age 13, cerebral magnetic resonance imaging, prompted by severe migraines, revealed focal hemosiderin deposits in the right temporal region, highly suggestive of focal intracerebral bleeding. On physical examination, approximately 50 widespread erythematous-violaceous macules and numerous firm nodules were observed on the limbs, trunk, and lower extremities (Fig 2), some of which were surgically excised. Histopathological analysis of a skin biopsy performed at age 19 revealed a diffuse dermal-hypodermal vascular proliferation consisting of thin-walled dilated vessels of varying size; some displayed slightly prominent (hobnail) endothelial cells, and others contained intraluminal papillary projections lined by endothelial cells (Fig 3, *A* and *B*). The endothelial cells were immunohistochemically positive for platelet endothelial cell adhesion molecule 1, marker of hematopoietic stem cells and hematopoietic progenitor cells (Fig 3, *C* and *D*) and focally for podoplanin/D2-40 but were negative for glucose transporter 1.

The clinical and histopathological features were retrospectively suggestive of multifocal lymphangiomatosis with thrombocytopenia. After discontinuation of all medications, the patient displayed no significant clinical or biological abnormalities during 22-year follow-up. He did not develop new cutaneous lesions and experienced no further episodes of GI bleeding. During the most recent clinical visit, his sole complaint related to disabling firm papules on the plantar arch, which were treated with bleomycin percutaneous sclerotherapy.

## Discussion

MLT is a multifocal vascular malformation characterized by the presence of numerous cutaneous reddish macules, papules, and nodules, with visceral involvement and thrombocytopenia attributed to platelet entrapment and consumption within dilated vascular lumina.^5^ While thrombocytopenia is typically a component of MLT, in some cases platelet counts have been normal.^5^ The most common sites of involvement in MLT are the skin, GI tract, lungs, CNS, musculoskeletal system, and peripheral nervous system.^3^

Given its rarity, the prevalence and pathogenesis of MLT remain unknown. This condition is often diagnosed within the first 6 months of life, frequently beginning with episodes of GI bleeding in the context of severe thrombocytopenia.^6^ Our patient exhibited widespread congenital cutaneous papules and macules, GI vascular lesions leading to severe GI bleeding starting in the neonatal period, and multifocal eye, bone, and CNS lesions, all accompanied by thrombocytopenia. Initial considerations included various diagnoses, especially Kasabach-Merritt syndrome related to Kaposiform hemangioendothelioma or tufted angioma. The retrospective diagnosis of MLT was established in adulthood at our National Referring Center for Superficial Vascular Anomalies, based on clinical features, thrombocytopenia, and pathological data. Compared to previously reported cases, our patient had ophthalmic involvement, broadening the spectrum of visceral involvement in MLT.

Currently, there is no standardized treatment for MLT. Treatment strategies aim to control the progression of vascular lesions, but outcomes vary.^7^^,^^8^ Treatment options include systemic corticosteroids, antiplatelets (aspirin or ticlopidine), vincristine, interferon-alpha 2A, thalidomide, propranolol, aminocaproic acid, octreotide, bevacizumab, and more recently sirolimus, which appears to be currently the more efficient therapeutic option.^3^^,^^9^ However, it remains unclear whether improvement of lesions in reported cases was related to treatment or the natural course of MLT. Our patient was managed with endoscopic interventions, corticosteroids and interferon, resulting in the complete resolution of GI bleeding and thrombocytopenia.

The prognosis of patients with MLT is typically poor, with a high mortality rate, particularly in early infancy (65%). Deaths are primarily attributed to CNS or GI tract lesions.^4^ Despite recurrent GI bleeding in childhood, our patient has been doing well, with no recurrence of bleeding or thrombocytopenia for at least 10 years of follow-up. Further cases should be studied to better understand the natural progression, genetic basis, and treatment of MLT.

MLT is a rare multiorgan vascular disorder with a generally poor prognosis. To our knowledge, our patient is the first case of MLT reported in the literature with a favorable long-term outcome into adulthood. However, it is difficult to ascertain whether the clinical and laboratory improvement in our patient resulted from treatment, spontaneous regression of the condition, or a combination of both.

## Conflicts of interest

None disclosed.
